# Heterologous Expression of Biopreservative Bacteriocins With a View to Low Cost Production

**DOI:** 10.3389/fmicb.2018.01654

**Published:** 2018-07-26

**Authors:** Beatriz Mesa-Pereira, Mary C. Rea, Paul D. Cotter, Colin Hill, R. Paul Ross

**Affiliations:** ^1^Teagasc Food Research Centre, Teagasc Moorepark, Fermoy, Cork, Ireland; ^2^APC Microbiome Ireland, University College Cork, Cork, Ireland; ^3^School of Microbiology, University College Cork, Cork, Ireland; ^4^College of Science Engineering and Food Science, University College Cork, Cork, Ireland

**Keywords:** bacteriocins, heterologous expression, *E. coli*, vectors, strains

## Abstract

Bacteriocins, a heterogenous group of antibacterial ribosomally synthesized peptides, have potential as bio-preservatives in in a wide range of foods and as future therapeutics for the inhibition of antibiotic-resistant bacteria. While many bacteriocins have been characterized, several factors limit their production in large quantities, a requirement to make them commercially viable for food or pharma applications. The identification of new bacteriocins by database mining has been promising, but their potential is difficult to evaluate in the absence of suitable expression systems. *E. coli* has been used as a heterologous host to produce recombinant proteins for decades and has an extensive set of expression vectors and strains available. Here, we review the different expression systems for bacteriocin production using this host and identify the most important features to guarantee successful production of a range of bacteriocins.

## Introduction

Bacteriocins are antimicrobial peptides produced by Gram-negative and Gram-positive bacteria. These molecules have attracted considerable interest, especially those produced by GRAS (Generally Recognized As Safe) microorganisms, as natural food preservatives in the food industry. They also represent potential alternatives to traditional antibiotics in the treatment of infections in humans and food-producing animals (Cotter et al., 2005; Desriac et al., 2010; Svetoch and Stern, 2010) and, in some cases, show promise as drugs for cancer treatment (reviewed in Kaur and Kaur, 2015).

Since the identification of colicin in 1925 (Gratia, 1925), many bacteriocins have been described (BAGEL4 database, http://bagel4.molgenrug.nl). However, only nisin (e.g., Nisaplin®) and pediocin PA-1 (e.g., ALTA®-2431) have been commercialized to any significant extent and these mainly as food biopreservatives. This may be explained by the multitude of studies that are required before a bacteriocin can be considered commercially viable, including characterization, potent antimicrobial activity, product stability, mechanism of action, mode of delivery, toxicity and assessment of their applications and industrial-scale production (Ingham and Moore, 2007).

The most important prerequisite for these studies is the production of high levels of biologically active bacteriocin. Although these peptides can be purified from their native producing strains, the process is time-consuming and laborious, and bacteriocin yields are often low (Rodríguez et al., 2003). Synthetic production is one alternative in some cases, but the complexity of some of the bacteriocins and the cost of the process limit the synthesis of large quantities (Chen et al., 2012). Therefore, attempts to increase the production of bacteriocins using alternative hosts such as *Lactococcus lactis* and other lactic acid bacteria (LAB) have been investigated (reviewed in Cintas et al., 2011). These strains, which are food-grade organisms, offer a safer choice for industrial food products, and provide the genetic and secretory machinery for efficient LAB bacteriocin production. However, the expression strains available are highly specific and the results in terms of yield are still disappointing at industrial level, restricting the variety and quantity of bacteriocin produced (Rodríguez et al., 2003). Given these limitations *E. coli*, the most commonly used organism for heterologous protein production, is an attractive option for the heterologous bacteriocin expression due to its rapid growth on inexpensive media, its extensive genetic characterization and the availability of versatile cloning tools, expression systems and strains (Mergulhao et al., 2004; Rosano and Ceccarelli, 2014; Jia and Jeon, 2016). This could facilitate the functional characterization and establishment of a production process in bacteriocins from sources that are difficult to cultivate, in addition to those bacteriocins discovered by data mining from sequenced bacterial genomes (Kuo et al., 2013), increasing their potential for manufacture and commerialization by food and pharmaceutical industries (Ongey and Neubauer, 2016). However, this approach is not without obstacles which may arise during the expression, secretion or processing of these peptides in *E. coli* (Choi and Lee, 2004).

In order to provide a guide for a design of a successful expression system for bacteriocin production in this host, the present review focuses on the different systems currently available for bacteriocin production in *E. coli*.

## General considerations before the expression system selection

### Bacteriocin

The choice of an expression system is not simple. Firstly, the characteristics of the bacteriocin should be taken into account, such as the presence of post-translational modifications and disulfide bonds, because these may affect its heterologous production.

According to Cotter et al. (2012), LAB bacteriocins are classified into those which are either post-translationally modified (class I) and unmodified or minimally modified peptides (class II). Class I can be subdivided into lantibiotics (with lanthionine bridges), linaridins, proteusins, linear azole-or azoline-containing peptides, cyanobactins, thiopeptides, lasso peptides, sactibiotics (contain sulfur-α-carbon linkages), bottromycins, glycocins, and modified microcins that do not belong to other subgroups. Class II is further divided into class IIa (the pediocin-like bacteriocins), IIb (the two-peptide bacteriocins), IIc (circular bacteriocins), IId (unmodified, linear, non-pediocin-like, single-peptide bacteriocins that do not belong to other subclasses), and IIe (the microcin E492-like bacteriocins). In this classification, the large (>10 kDa), heat-labile antimicrobial peptides bacteriolysins (formerly class III bacteriocins) were removed from the bacteriocin category.

Similarly, bacteriocins from Gram-negative organisms can be divided into small peptides, such as microcins [class I (presence of modifications) or II (unmodified)], and large peptides such as colicins (Drider and Rebuffat, 2011).

### Genes required for bacteriocin expression

In general, the production of bacteriocins in the native host requires several genes including a structural gene that encodes the prepeptide (or two structural genes for the two peptide bacteriocins). Other genes encode an immunity protein, specialized secretion machinery and in many cases proteins capable of performing modifications and regulatory sequences (Nes et al., 1996). Therefore, different strategies are required depending on the characteristics of each bacteriocin to ensure production (see Table 1).

**Table 1:** Bacteriocins heterologously produced by *E. coli*.

**Bacteriocin**	**Native host^a^**	***E. coli* strain^b^**	**Vector^c^**	**Location^d^**	**Culture conditions^e^**	**References**
Ala(0)actagardine (Class I, Lantibiotic)	*A. garbadinensis* ATCC 31049	BL21 (DE3)	pRSFDuet-1-*garA*-*garM* pCDFDuet-1-2X*garO*	SCF	LB, 0.2 mM IPTG 20 h, 18°C	Shi et al., 2012
BacR1 (Class II)	*Sta. aureus* UT0007	BL21 (DE3)	pSuV1- bacR1^*^^c^	M	LB, 2 mM IPTG ON, RT	Ingham et al., 2005
Bactofencin A (Class IId)	*Lb. salivarius* DPC6502	Tuner (DE3)	pETcoco-2-*bfnAbfnI* *DLSL0052DLSL0053*	M	LB, 0.05–0.1 mM IPTG 3 h, 37°C	Mesa-Pereira et al., 2017
Bovicin HJ50 (Class I, Lantibiotic, SS bond)	*Str. bovis* HJ50	BL21 (DE3)	pET28a-*bovAM*, pET28a-*bovT150*	SCF	BovA expression: LB, 0.5 mM IPTG 20 h, 18°C BovT150 expression: LB, 0.5 mM IPTG 12 h, 20°C	Lin et al., 2011
	*Str. bovis* HJ50	BL21 (DE3)	pET28a-*bovAM* pACYC-Duet-*bovT* or *bovT_150_*	SCF	LB, 0.5 mM IPTG 5, 10, 20 h, 37°C	Wang et al., 2016
	*Str. bovis* HJ50	C43 (DE3)	pET28a-*bovAM* pACYC-Duet-*bovT* or *bovT_150_*	M	LB, 0.5 mM IPTG 10-20 h, 37°C	Wang et al., 2016
Carnobacteriocin B2 (Class IIa)	*C. piscicola* LV17B	–	pMALc-CbnB2^*^ pMALc-*CbnB2P*	TE	Rich broth, 0.3 mM IPTG 3 h, 37°C	Quadri et al., 1997
	*C. maltaromaticum* CP5	BL21 (DE3)	pET32a-CbnB2^*^	TE	TB, 0.55 mM IPTG or 14.6 mM lactose 3 h, 37°C	Jasniewski et al., 2008
Carnobacteriocin BM1 (Class IIa)	*C. maltaromaticum* CP5	BL21 (DE3)	pET32a-CbnBM1^*^ pET32a-CbnBM1M41V^*^	TE	TB, 0.55 mM IPTG or 14.6 mM lactose 3 h, 37°C	Jasniewski et al., 2008
ColA-43864 (Colicins)	*Cit. freundii* ATC43864	S17-1	pMQ124-*colA-43864*	SCF	LB, 0.2% L-arabinose 3 h, 37°C	Shanks et al., 2012
Colicin V (Colicins)	*E. coli*	KS300/pMS421	pHLZ01 (pBR322)- cvaC^*^	TE (periplasm)		Zhang et al., 1995
Divercin AS7 (Class IIa, two SS bonds)	*C. divergens* AS7	BL21 (DE3) pLys	pET28b-*AS7*	SCF	LB, 0.1 mM IPTG 24 h, 37°C	Olejnik-Schmidt et al., 2014
Divercin V41 (Class IIa)	*C. divergens* V41	Origami (DE3) pLysS	pET32b- DvnV41^*^c	SCF	TB, 1 mM IPTG 3 h, 37°C	Richard et al., 2004
	*C. divergens* V41	BL21 (DE3)	pSuV1- DivV41^*^^c^	M	SOC, 2 mM IPTG ON, RT	Ingham et al., 2005
	*C. divergens* V41	Origami (DE3) pLysS/pCR03	pET32b- DvnV41^*^	SCF	TB or M9, 0-2 mM IPTG 3 h, 30°C	Yildirim et al., 2007
Divergicin A (Class IIc)	*C. divergens LV13*	MC4100/pHk22	pMG36e-Leucocin A leader-divergicin A pMG36e-ColV leader-divergicin A	M	YT and 0.2mM 2,2′-dipyridyl. 37°C	Van Belkum et al., 1997
	*C. divergens LV13*	BL21 (DE3)/pHk22	pT7-1-Lactococcin A leader-divergicin A	TE	YT and 0.2 mM 2,2′-dipyridyl, 0.4 mM IPTG 2 h, 37°C	Van Belkum et al., 1997
E50-52 (Class IIa)	*Ent. faecium* NRRL B-30746	BL21 (DE3)	pET SUMO-*rb50-52*	SCF	LB, 1.5 mM IPTG 5 h, 37°C	Wang et al., 2013
Enterocin A (Class IIa)	*Ent. faecium* ATB 197a	BL21 (DE3)	pET37b-*entA*	TE SCF M	LB, 0.1 mM IPTG 1-4 h, 37°C	Klocke et al., 2005
Enterocin B (Class II)	*Ent. faecium* ATB 197a	BL21 (DE3)	pET37b-*entB*	TE	LB, 0.1 mM IPTG 1–4 h, 37°C	Klocke et al., 2005
Enterocin CRL35 (Class IIa, one SS bond)	*Ent. mundtii* CRL35	Rosetta (DE3) pLysS	pET22b-*munA*	M	LB or M9, 0.5 mM IPTG 1 h, 37°C	Masias et al., 2014
	*Ent. mundtii* CRL35	BL21 (DE3) pLysS	pET22b-*munA*	M	LB, 0.5 mM IPTG 1 h, 37°C	Masias et al., 2014
	*Ent. mundtii* CRL35	C41 (DE3) pLysS	pET22b-*munA*	M	LB, 0.5 mM IPTG 1 h, 37°C	Masias et al., 2014
	*Ent. mundtii* CRL35	C43 (DE3) pLysS	pET22b-*munA*	M	LB, 0.5 mM IPTG 1 h, 37°C	Masias et al., 2014
	*Ent. mundtii* CRL35	Rosetta –gami 2 (DE3)	pET22b-*munA*	M	LB, 0.5 mM IPTG 1 h, 37°C	Masias et al., 2014
	*Ent. mundtii* CRL35	Origami (DE3)	pET22b-*munA*	M	LB, 0.5 mM IPTG 1 h, 37°C	Masias et al., 2014
	*Ent. mundtii* CRL35	BL21 (DE3)	p8760 (PBAD24)-EtpM-*munA* pACYCDuet-1-*munC*	TE (membrane)	Ent35: LB, 0.6% arabinose 5 min MunC: LB, 0.02% lactose 1 h, 37°C	Barraza et al., 2017
Enterocin P (Class IIa)	*Ent. faecium*	BL21 (DE3)	pTYB1-EntP^*^^c^	M	LB, 2 mM IPTG ON, RT	Ingham et al., 2005
	*Ent. faecium* P13	Tuner (DE3) pLacI	pETBlue-1-*entP* pETBlue-1*-entP-entiP*	M SCF IB	M9, 0-1 mM IPTG 3 h, 37°C	Gutiérrez et al., 2005
Epidermicin NI01 (Class IId)	*Sta. epidermidis* 224	BL21 (DE3)	pET29a-*edcA*	SCF	2 × YT broth, 0.05 mM IPTG ON, 17°C	Sandiford and Upton, 2012
Gassericin A (class IIc)	*Lb. gasseri* LA39	JM109	PinPoint Xa-1-gassericinA^*^	SCF	LB, 2 μmol biotin 8 h, 37°C	Kawai et al., 2003
Haloduracin (Class I, two-peptide lantibiotic)	*B. halodurans*	BL21 (DE3)	pRSFDUET-1-*HalA1*-Xa-*HalM1* pRSFDUET-1-*HalA2*-Xa-*HalM2*	SCF	LB, 0.5 mM IPTG 18 h, 18°C	Shi et al., 2011
Lactococcin G (Class IIb, two-peptides)	*L. lactis* LMGT 2081	BL21 RIL (DE3) pLysS	pGEV-*LcnG-α* or *LcnG-*β	IB	M9, 1 mM IPTG 6-8 h, 37°C	Rogne et al., 2008
Lactococcin K	*L. lactis* MY23	BL21 (DE3)	pEMBP- *lcnK* pET21c- *lcnK*	SCF IB	LB, 1 mM IPTG 12 h, 37°C	Kim et al., 2006
Lichenicidin (Class I, two-peptide lantibiotic)	*B. licheniformis* 189	BLic5	pCC2FOS™- *lic* operon	M	Medium M 24 h, 37°C	Caetano et al., 2011a,b
	*B. licheniformis* 189	BL21 gold (DE3)	pRSFDuet-1_*TM1A1* and pRSFDuet-1_*TPM2A2*	M	Medium M, 2 × YT, SB, TB and LB-Kelly 0.5 mM IPTG 24 h, 30°C	Kuthning et al., 2015
LSEI_2163 (Class IId, one SS bond)	*Lb. casei* ATCC334	Origami (DE3) pLysS	pAB-238- *LSEI_2163*	TE	LB, 1 mM IPTG 5 h, 37°C	Kuo et al., 2013
LSEI_2386 (Class II)	*Lb. casei* ATCC334	Origami (DE3) pLysS	pAB-238- *LSEI_*2386	TE	LB, 1 mM IPTG 5 h, 37°C	Kuo et al., 2013
Mersadicin (Class I, Lantibiotic)	*B. licheniformis* MKU3	M15/pRep4	pQE-30UA-*lanA*	SCF	LB, 0.4 mM IPTG 4 h, 37°C	Kayalvizhi et al., 2016
Mesentericin Y105 (Class IIa)	*Leu. mesenteroides* Y105	DH5α	pBluescript SKII+- *dvnA* leader- mesY^*^-*mesI* pBluescript SKII+- *dvnA* leader- *mesY*-*mesI*	M	LB, 1 mM IPTG 37°C	Biet et al., 1998
Microcin B	*Ps. syringae* pv. *glycinea* B076	BL21 (DE3)	pBAD His/B- *mcb*	SCF	M9, 10 mM Arabinose 24 h, 30°C	Metelev et al., 2013
Nisin (Class I, Lantibiotic)	*L.lactis*	BL21 (DE3)	pRSFDUET-1-*nisA*- *nisB* pACYCDUET-1-*nisC*	SCF	LB, 0.5 mM IPTG 15 h, 18°C	Shi et al., 2011
Nukacin ISK-1 (Class I, Lantibiotic)	*Sta. warneri* ISK-1	BL21 (DE3)	pET14b-*nukAM*	SCF	2 xYT, 1 mM IPTG 20 h, 20°C or 3 h, 37°C	Nagao et al., 2005
Pediocin AcH (Class IIa, two SS bonds)	*P. acidilactici* H	E609L	pPR682-pap^*^	M	LB, 1 mM IPTG 3 h, 37°C	Miller et al., 1998
	*P. acidilactici* LB42-923	JM109	pHPS9- *pap* operon	M	LB, 37°C	Bukhtiyarova et al., 1994
Pediocin PA-1 (Class IIa, two SS bonds)	*P. acidilactici* PAC1.0	V850	pSRQ11- *ped* operon pSRQ11.2- *ped* operon pBR322- *ped* operon	M	M9 supplemented with 1% yeast extract and 1% Hy Case ON, 37°C	Marugg et al., 1992
	*P. acidilactici* F	DH5α	pPC418- *ped* operon pHPS9- *ped* operon	M	LB, ON, 37°C	Coderre and Somkuti, 1999
	*P. acidilactici*	BL21 (DE3)	pSuV1- PedPA-1^*^^c^	M	LB, 2 mM IPTG ON, RT	Ingham et al., 2005
	*P. acidilactici* K10	M15/pRep4	PQE-30 Xa-pedA^*^	ET	LB, 1 mM IPTG 4 h, 37°C	Moon et al., 2005
	*P. acidilactici* K10	M15/pRep4	PQE-40-pedA^*^	ET IB	LB, 1 mM IPTG 4 h, 37°C	Moon et al., 2006
	*P. acidilactici* PAC1.0	Origami (DE3)	pET32b- pedA^*^	SCF	LB, 0.02 mM IPTG 4 h, 37°C	Beaulieu et al., 2007
	*P. acidilactici* PA003	BL21 (DE3)	pET32b- pedA^*^ pET20b- pedA^*^	IB SCF	LB, 0.02 mM IPTG 4 h, 37°C	Liu et al., 2011
	*P. acidilactici* LMG2351	Tuner (DE3)	pETcoco2-*ped* operon	M	LB, 0.05-0.1 mM IPTG 3 h, 37°C	Mesa-Pereira et al., 2017
Piscicolin 126 (Class IIa, one SS bond)	*C. piscicola* JG126	AD494 (DE3)	pET32- pisA^*^	TE	LB, 0.1 mM IPTG 4 h, 37°C	Gibbs et al., 2004
	*C. piscicola*	BL21 (DE3)	pSuV1- pisA^*^^c^	M	LB, 2 mM IPTG ON, RT	Ingham et al., 2005
Plantaricin E (Class IIb, two-peptide bacteriocin)	Soil metagenome	BL21 (DE3)	pET32a-plnE^*^	SCF	LB, 1 mM IPTG 5 h, 16, 25, 30 and 37°C	Pal and Srivastava, 2014
	*Lb. plantarum* LR/14	BL21 (DE3)	pET32a-*plnE,plnG* or *plnH*. pET28a-*plnE,plnG* or *plnH*.	SCF IB (PlnG and PlnH)	LB, 1 mM IPTG 16 h, 22°C	Pal and Srivastava, 2015a
	Soil metagenome	BL21 (DE3)	pET32a-*plnE*	SCF	Small scale: LB, TB 0.5 mM IPTG 5 and 9 h at 22°C Large scale: 12 L LB 0.5 mM IPTG 3 h, 25 and 30°C	Pal and Srivastava, 2015b
	*Lb. plantarum* 163	BL21 (DE3)	pET32a-plnEm^*^	SCF	LB, 0.5 mM IPTG 8 h, 25°C	Meng et al., 2017
Plantaricin EF (Class IIb, two-peptide bacteriocin)	*Lb. plantarum* C11	BL21 RIL (DE3) pLysS	pGEV2- *plnE* pGEV2- *plnF*	IB	M9, 1 mM IPTG 4 h, 37°C	Fimland et al., 2008
	*Lb. plantarum*	BL21 (DE3)	pET32a-*plnE* pET32a-*plnF*	SCF	LB, 1 mM IPTG 6 h, 16, 20, 25, 30, 37°C	Tang et al., 2018
Plantaricin F	Soil metagenome	BL21 (DE3)	pET32a-plnF^*^	SCF	LB, 1 mM IPTG 5 h, 16, 25, 30 and 37°C	Pal and Srivastava, 2014
Plantaricin J	Soil metagenome	BL21 (DE3)	pET32a-plnJ^*^	SCF	LB, 1 mM IPTG 5 h, 16, 25, 30 and 37°C	Pal and Srivastava, 2014
Plantaricin JK (Class IIb, two-peptides)	*Lb. plantarum* C11	BL21 RIL (DE3) pLysS	pGEV2- *plnJ* pGEV2- *plnK*	IB	LB or M9, 1 mM IPTG 3-4 h, 37°C	Rogne et al., 2009
Plantaricin K	Soil metagenome	BL21 (DE3)	pET32a-plnK^*^	SCF	LB, 1 mM IPTG 5 h, 16, 25, 30 and 37°C	Pal and Srivastava, 2014
Plantaricin NC8 (Class IIb, two-peptide bacteriocin)	*Lb. plantarum* ZJ316	BL21 (DE3)	pET32a- *PLNC8α* pET32a- *PLNC8β*	SCF	LB 0.1, 0.2, 0.5, 1 mM IPTG 5,10,16, 20 h 16, 20, 25, 30 and 37°C	Jiang et al., 2016
Plantaricin Pln1 (Class II)	*Lb. plantarum* 163	BL21 (DE3)	pET32a-*pln1*	TE	LB 0.5, 1, 2, 4 mM IPTG 4, 6, 8, 10 h 20, 25, 30 and 37°C	Meng et al., 2016
Plantaricin S34 (Class II)	*Lb. plantarum* S34	BL21 (DE3) pLysS	pET32a-plnF^*^ pET32a-plnE^*^	SCF	LB, 0.5 mM IPTG 5 h, 22°C	Mustopa et al., 2016
Prochlorosin 1.7, 2.11 and 3.3 (Class I, Lantibiotics)	*Prochlorococcus*	BL21 (DE3)	pRSFDUET-1-*procA*-*procM*	SCF	LB, 0.1 mM IPTG 20 h, 18°C	Shi et al., 2011
Pyocin S4	*Ps. aeruginosa* PAO	BL21 (DE3) pLysS	pET15b-S4imm	SCF	LB, 1 mM IPTG ON, 28°C	Elfarash et al., 2012
Sakacin P (class IIa)	*Lb. sakei*	BL21 (DE3)	pET28a–sakP^*^	IB	LB, 0.8 mM IPTG 3 h, 20 or 37°C	Chen et al., 2012
Subtilosin A (Sactipeptide)	*B. subtilis* 168	BL21 (DE3)/pPH151	pETDuet-*sboA*-*albA*	IB	LB, 0.5 mM IPTG 22–24 h, 18°C	Himes et al., 2016
Suicin (Lantibiotic, SS-bond)	*Str. suis* serotype 2	BL21 (DE3)	pET28a-*suiAM* pET28a-*suiTR*	IB	LB, 0.5 mM IPTG 20 h 16°C	Wang et al., 2014
Warnericin RK (Class II)	*Sta. warneri* RK	M15/pREP4	pQE30-*war^*c*^* pQE70-*war^*c*^*	TE	LB or M9, 1 mM IPTG 6 h, 37°C	Verdon et al., 2013

a *Native hosts: A., Actinoplanes; B., Bacillus; C., Carnobacterium; Cit., Citrobacter; E., Escherichia, Ent., Enterococcus; Str., Streptococcus; L., Latococcus; Lb., Lactobacillus; Ps., Pseudomonas; Sta., Staphylococcus; P., Pediococcus*.

b *E. coli strains containing plasmids: pCR03, derivative pET-32 plasmid; pHK22, contains the structural gene and the immunity gene for colicin V as well as the genes encoding the two inner-membrane transport proteins, CvaA and CvaB, for colicin V; pMS421, pSC101 with lacI^Q^; pPH151, containing the E. coli suf ABCDSE genes that facilitate the proper assembly and repair of the Fe– S cluster; pREP4, contains lacI gene for regulating expression from PQE vectors. pLysS and pLacI information is listed in the text*.

^c*^ Mature sequence (without leader peptide),

c *codon optimized genes*.

d *Location: IB, Inclusion bodies; SCF, Soluble Cellular Fraction (soluble fraction after cell pellet sonication); M, culture medium (cell-free supernatants), TE, Total cell extract*.

e *Culture medium: Luria broth (LB) medium, Terrific broth (TB), 2 × Yeast extract-Tryptone broth (2 × YT). ON, overnight; RT, Room Temperature*.

In most cases, the expression of the structural gene or its mature sequence is enough to produce the active bacteriocin. Some examples include carnobacteriocin B2 (Jasniewski et al., 2008), divercin AS7 and V41(Richard et al., 2004; Ingham et al., 2005; Yildirim et al., 2007; Olejnik-Schmidt et al., 2014), epidermicin NI01 (Sandiford and Upton, 2012), gassericin A (Kawai et al., 2003), or sakacin P (Chen et al., 2012) (see Table 1). However, the transporter gene is also necessary for the synthesis of some bacteriocins, for example in the production of pediocin PA-1 and bactofencin A (Bukhtiyarova et al., 1994; Mesa-Pereira et al., 2017). In other cases, the co-expression of the structural gene with the genes involved in post-translational modifications on the same or different plasmids are required for the heterologous expression of lantibiotics such as lichenicidin (Caetano et al., 2011a,b; Kuthning et al., 2015), nukacin ISK-1 (Nagao et al., 2005), prochlorosin, haloduracin, nisin (Shi et al., 2011), suicin (Wang et al., 2014), and the sactibiotic subtilosin A (Himes et al., 2016), amongst others.

### Toxicity

The potential toxicity to *E. coli* due to the overexpression of the mature peptides or components of the secretion machinery and other bacterial integral membrane proteins (Fath and Kolter, 1993; Miller et al., 1993) must also be considered as these could interfere with the growth and viability of *E. coli*, limiting bacteriocin production (Bentley et al., 1990; Mccormick et al., 1996; Biet et al., 1998; Gutiérrez et al., 2005; Ingham et al., 2005; Moon et al., 2005; Masias et al., 2014; Mesa-Pereira et al., 2017).

## Plasmids for bacteriocin expression in *E. coli*

### General features

**Figure 1:**
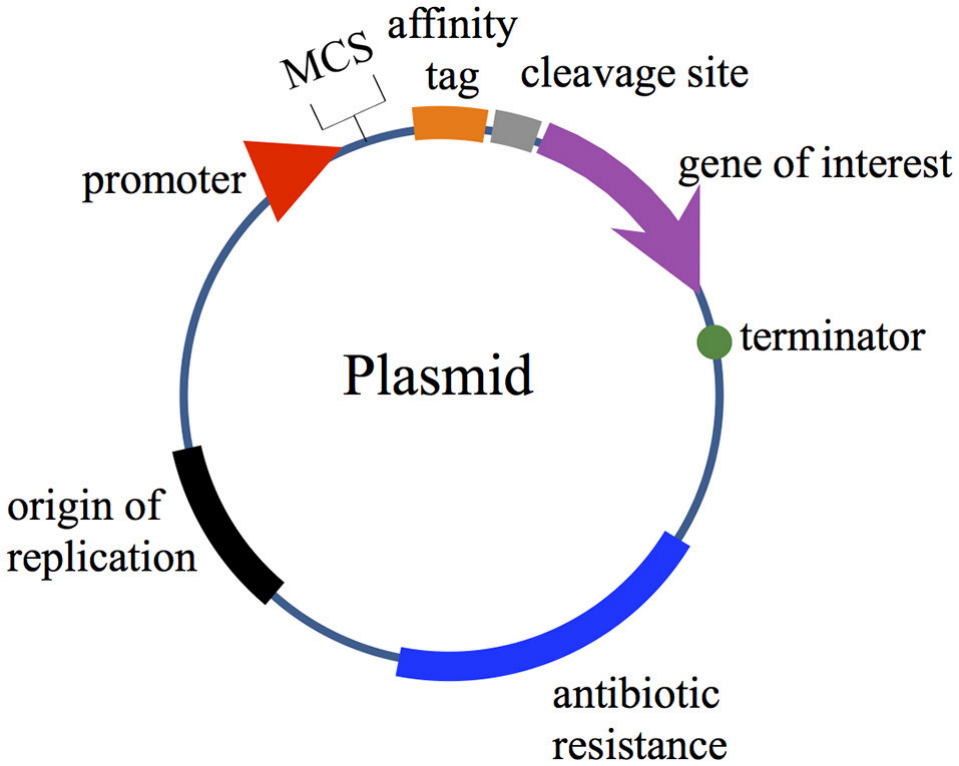
Schematic plasmid map showing the major features present in common expression vectors.

Expression vectors require various components to carry out their functions, including; (i) an origin of replication; (ii) a selection marker (generally genes encoding resistance to antibiotics); (iii) a promoter region for gene transcription initiation; and (iv) multiple unique restriction enzyme sites arranged in a polylinker region after the promoter to facilitate the cloning (referred to as multiple cloning sites; MCS). In some cases, two or more MCS are available in commercial plasmids (i.e., Duet vectors and pRSFDuet™-1) for cloning several genes of interest without the need to use multiple plasmids. In addition, the MCS can additionally provide fusion tags to facilitate the purification of the expressed bacteriocins. Plasmids must also contain one or several terminators to ensure an efficient transcriptional termination and prevent the transcription downstream of the coding sequence of interest (Figure 1). In terms of translational features, the plasmid must include a ribosome binding site (RBS) with a Shine-Dalgarno sequence (UAAGGAGG) located 5–13 bases upstream of the start codon for the interaction with the 3′ end of rRNA during translation initiation (reviewed in Mergulhao et al., 2004; Terpe, 2006; Durani et al., 2012; Rosano and Ceccarelli, 2014).

### Plasmid copy number

A replicon consists of one origin of replication with associated control elements and is involved in plasmid replication and copy number control (del Solar and Espinosa, 2000). The number of copies per cell can vary between one and approximately one hundred. Theoretically, the higher the copy number of a plasmid, the higher the expression of the gene of interest. However, this often results in aggregation, misfolding or protein degradation in *E. coli* (Tolia and Joshua-Tor, 2006), and could also cause cellular toxicity when the overexpression of secretion machinery and other integral membrane proteins are required for bacteriocin production. For this reason, low and middle copy vectors (15–20 copies per cell) based on ColE1, pMB1 replicons, including the pBR322 and pET vector systems and pACYC plasmids with an origin replication derived from p15A, have been successfully used for bacteriocin expression (Table 2). In addition, the use of two plasmids with compatible origins (e.g., ColE1 with p15A, pMB1 with p15A) has allowed for dual expression of proteins for functional characterization (Table 1). In this regard, the expression vector pETcoco-2 could be a useful tool for studying whole operons since it enables the control of copy number by arabinose induction, facilitating the optimization of bacteriocin expression (Sektas and Szybalski, 2002; Mesa-Pereira et al., 2017).

**Table 2:** Features of expression vectors used for bacteriocin production in *E.coli*.

**Vector^a^**	**Size (bp)**	**Promoter^b^**	**Selection^c^**	**Tags and fusion partners^d^**	**Protease cleavage sites^e^**	**Origin**	**Supplier/References**
pAB-238	5,800	T7*lac*	Amp	N-Trx	Thr	pBR322	Kuo et al., 2013
pACYCDuet-1^*^	4,008	T7*lac*	Cm	N-His S	None	P15A	Novagen
pBAD His/B	4,100	*araBAD*	Amp	N-His	EK	pUC	Invitrogen
pBAD-24	4,542	P*_*BAD*_*	Amp	None	None	pBR322	Invitrogen
pBluescript SKII+	2,961	*lac*	Amp	None	None	pUC	Stratagene
pBR322	4,361	None	Amp, Tet	None	None	pMB1	NEB
pCC2FOS™	8,181	T7	Cm	None	None	*oriV*/*oriS*	Epicentre Biotechnologies
pCDFDuet-1^*^	3,781	T7*lac*	Str/Spe	N-His S	None	pCloDF13	Novagen
pEMBP		T7	Amp	MBP	EK	pBR322	Bioprogen
pETBlue-1	3,476	T7*lac*	Amp	C-His	None	pUC	Novagen
pETcoco-2	12,417	T7lac pBAD	Amp	N-His S C-HSV tag	EK	Mini-F/RK2	Novagen
pETDuet-1	5,420	T7	Amp	N-His S	None	pBR322	Novagen
pET SUMO	5,643	T7*lac*	Kan	N-His N-SUMO	SUMO protease	pBR322	Invitrogen
pET-14b	4,671	T7	Amp	N-His	Thr	pBR322	Novagen
pET-15b	5,708	T7	Amp	N-His	Thr	pBR322	Novagen
pET-20b (+)	3,716	T7	Amp	Signal sequence C-His	None	pBR322	Novagen
pET-21c	5,441	T7*lac*	Amp	C-His	None	pBR322	Novagen
pET-22b (+)	5,493	T7*lac*	Amp	Signal sequence C-His	None	pBR322	Novagen
pET-28a,b	5,369	T7*lac*	Kan	N-His C- His	Thr	pBR322	Novagen
pET-29a	5,371	T7*lac*	Kan	C-His Stag	Thr	pBR322	Novagen
pET-32a,b	5,900	T7*lac*	Amp	N-Trx Internal His C-His	Thr EK	pBR322	Novagen
pET-37b (+)	–	T7*lac*	Kan	Signal sequence N-CBD*_*cenA*_* C-His	Thr Xa	pBR322	Novagen
pGEV2	>5,443	T7*lac*	Amp	N-GB1 domain C-His	Thr Xa	pBR322	Huth et al., 1997
PinPoint	3,331	T7 or *tac*	Amp	Biotin	Xa	ColE1	Promega
pHPS9^**^	5,700	P59	Em Cm	None	None	pMB1 pTA1060	ATCC
pMG36e	3,700	P32	Em	None	None	pWV01	van de Guchte et al., 1989
pMQ124	7,621	P*_*BAD*_*	Gm	None	None	ColE1/pRO1600	Shanks et al., 2009
pPC418^**^	9,135	STP_2201_	Amp Em	None	None	–	Coderre and Somkuti, 1999
pPR682 (pMAL-c2x)	6,645	*tac*	Amp	N-MBP	Xa	ColE1	NEB
pQE-30 UA	3,504	T5*lac*	Amp	N-His	None	ColE1	Quiagen
pQE-30 Xa	3,500	T5*lac*	Amp	N-His	Xa	ColE1	Quiagen
pQE-40	4,031	T5*lac*	Amp	N-His N-DHFR	None	ColE1	Quiagen
PQE-70	3,426	T5*lac*	Amp	C-His	None	ColE1	Quiagen
pRSFDuet-1^*^	3,829	T7*lac*	Kan	N-His S	None	RSF1030 (NTP1)	Novagen
pT7-1	2,400	T7	Amp	None	None	ColE1	Tabor and Richardson, 1985
pTYB12	7,417	T7	Amp	N-VMA intein CBD	None	pBR322	NEB
pSRQ11^***^	9,400	–	Em	–	None	–	Gonzalez and Kunka, 1987
pSuV1	7,332	T7	Amp	*pelB* signal sequence C-VMA intein CBD	Self-cleavage	ColE1	Ingham et al., 2005

a* *Vectors containing two MCS (Multiple Cloning Site)*.

** *Shuttle vectors: pHPS9 E.coli-Bacillus subtilis shutle vector; pPC418, E. coli–St. thermophiles shuttle vector*.

*** *pSRQ11 PA-1 pediocin plasmid*.

b *Promoters information is listed in the text. constitutive p32 and p59 promoter from L. lactis subsp. cremoris Wg2, STP2201 promoter from S. thermophilus ST128*.

c *Antibiotic resistance markers: Amp, ampicillin; Cm, chloramphenicol; Em, erythromycin; Gm, gentamicin; Kan, kanamycin; Tet, tetracycline; Str, streptomycin; Spe, spectinomycin*.

d *CBD, chitin binding domain; DHFR, Dehydrofolate reductase; GB1 domain: immunoglobulin- DNA binding domain of streptococcal protein G*.

e *EK, enterokinase; Thr, thrombin*.

### Promoter region

A careful balance of promoter strength and gene copy number is necessary for the optimization of the bacteriocin expression level. Since bacteriocins could be toxic for the host, the promoter strength should be adequate in order to minimize the metabolic burden on *E. coli* prior to the production phase and prevent the saturation of the host transport machinery (Rosenberg, 1998; Mergulhão et al., 2003; Mergulhao et al., 2004). This might explain the failure to generate successful expression plasmids under the control of constitutive promoters (Mccormick et al., 1998; McCormick et al., 1999), while the use of inducible promoters enables stable bacteriocin expression (Gutiérrez et al., 2005). Thus, an appropriate promoter must be strong, have a low basal expression level (i.e., be highly repressible) and a cost-effective induction system.

A large number of promoter systems have been described for protein production in *E. coli* (reviewed in Terpe, 2006; Durani et al., 2012; Rosano and Ceccarelli, 2014). In terms of bacteriocin expression, the L-arabinose inducible *araBAD* (P_BAD_) promoter has been used in some studies (Shanks et al., 2012; Metelev et al., 2013; Barraza et al., 2017), while most bacteriocins have been expressed using *lac*-derived promoters inducible by lactose or its non-hydrolyzable analog isopropyl β-D-thiogalactopyranoside (IPTG), including *tac* promoter (Miller et al., 1998; Kawai et al., 2003), T5*lac* (Moon et al., 2005, 2006; Verdon et al., 2013; Kayalvizhi et al., 2016) and the most widely used T7 and T7*lac* promoters (Table 2).

In T7 promoter systems, the gene of interest is cloned behind a promoter recognized by the phage T7 RNA polymerase that is provided by another plasmid or in the bacterial genome in a prophage (λDE3). T7 RNA polymerase is under the transcriptional control of a *lac*UV5 promoter inducible by lactose or IPTG. Basal expression can be controlled by the introduction of a mutated promoter of the *lacI* gene, called *lacI*^Q^, that increases the expression of the *lac* promoter repressor LacI. Additionally, the co-expression of T7 lysozyme provided in a compatible plasmid (pLysS or pLysE) can inhibit the transcription of T7 RNA polymerase. In the case of the T7*lac* promoter, this also includes a *lac*O operator downstream of the promoter that avoids basal expression (reviewed in Rosano and Ceccarelli, 2014).

### Protein targeting-signal secretion sequences

Since *E. coli* is a Gram-negative bacterium, bacteriocin production can be confined to the cytoplasm where it can accumulate in a soluble form or aggregate in insoluble inclusion bodies, or can also be secreted into the periplasmic space or into the culture medium. Several factors including protein size, amino acid composition, and the type of leader peptide can affect bacteriocin translocation to these locations (reviewed in Mergulhao et al., 2004).

**Figure 2:**
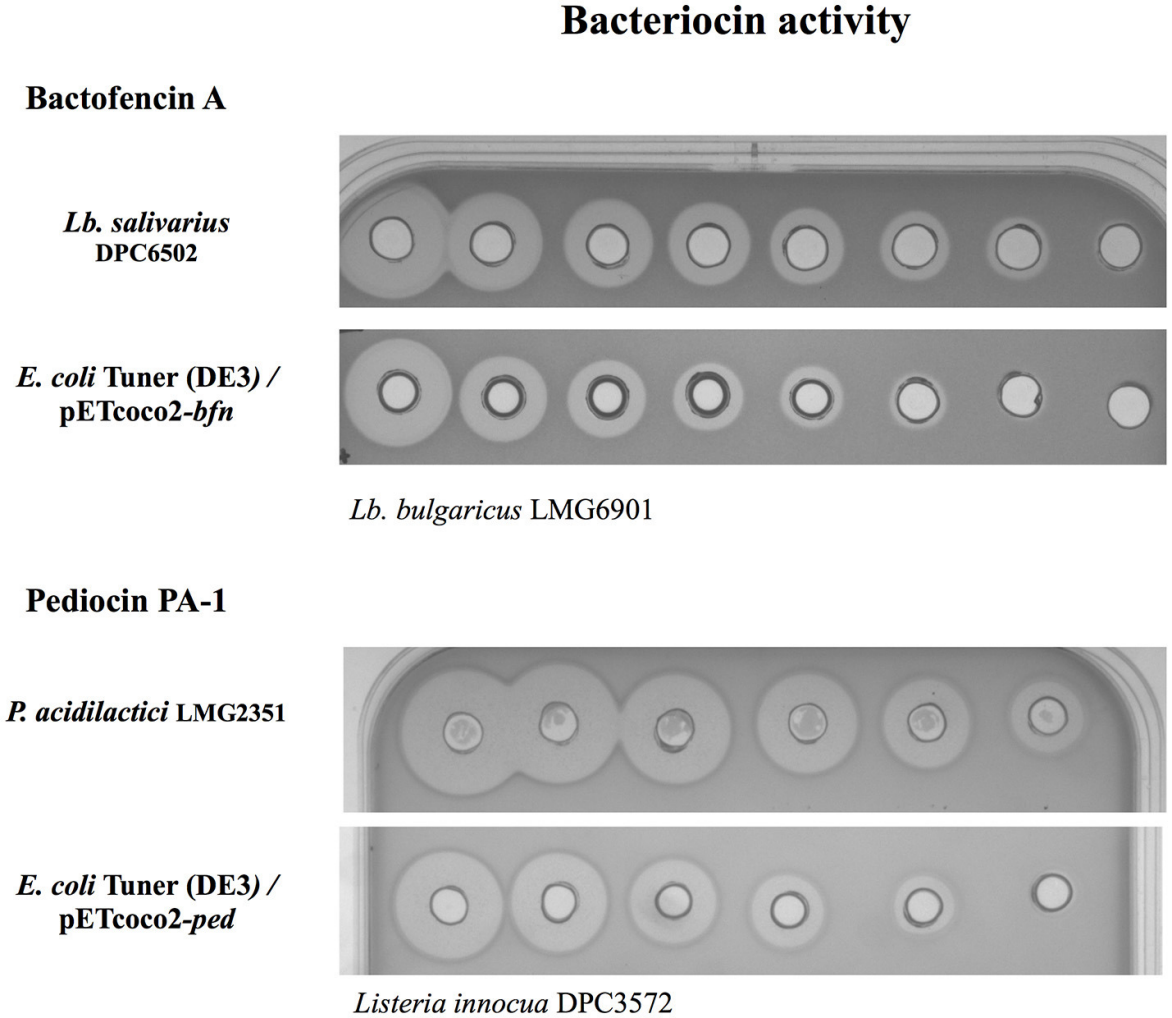
Bacteriocin activity of 2-fold serial dilutions of cell-free supernatants. Antimicrobial activity of bactofencin A against *Lb. bulgaricus* LMG 6901 produced by *Lb. salivarius* DPC6502 (natural producer) and *E. coli* Tuner (DE3) carrying the vector expressing the whole bactofencin A operon (*bfn*) and pediocin PA-1 against *Listeria innocua* DPC3572 produced by *P. acidilactici* LMG2351 (natural producer) or by *E. coli* Tuner (DE3) carrying the plasmid with the pediocin PA-1 operon (*ped*).

Most bacteriocins use a dedicated secretion machinery in their natural hosts to export the bacteriocin to the extracellular media. However, the production of active bacteriocins directly to the culture medium by cloning their whole operons in *E. coli* has been reported on a limited number of occasions (Figure 2) (Caetano et al., 2011a; Mesa-Pereira et al., 2017). Generally, the overexpression of cloned native genes leads to bacteriocin accumulation in inclusion bodies in the cytoplasm. At first glance, the formation of inclusion bodies could be advantageous as the expressed bacteriocins are inactive and protected against host proteases, facilitating their purification and a high protein yield. However, this aggregation can affect the host metabolism and additional steps (inclusion body isolation, solubilization of the aggregates, and protein refolding) are required for their purification, limiting their large-scale production (Mergulhao et al., 2004).

The processing and the correct folding of bacteriocins as well as their recovery can be simplified when the peptide is secreted into the *E. coli* periplasm or into the culture medium. To achieve that, signal sequences of proteins recognized by a general protein secretory pathway (*sec* pathway), such as the maltose-binding protein *malE* (Miller et al., 1998), the pectase lyase secretion signal *pelB* (Ingham et al., 2005), and the outer membrane protein *ompA* (Zhang et al., 1995), have been fused to the bacteriocin sequence for targeting their secretion to the periplasm. In addition, the bacteriocin divergicin A signal peptide has been used to direct the expression of mesentericin Y105 in *E. coli* in the absence of their dedicated secretion machinery (Biet et al., 1998). This relies on the fact that divergicin A, as well as other bacteriocins such as hiracin JM79 (Sánchez et al., 2007), acidocin B (Leer et al., 1995), and enterocin P (Cintas et al., 1997; Gutiérrez et al., 2005), can be exported by the *E. coli sec* pathway. Subsequently, the peptides can be secreted into the culture medium by osmotic shock or cell wall permeabilization or, alternatively, be released directly into the medium using a periplasmic leaky *E. coli* host (E609L, Miller et al., 1998).

It is important to mention that bacteriocins containing disulfide bonds are normally accumulated in the periplasm where disulfide binding proteins catalyze the oxidation process (Miller et al., 1998) or alternatively overexpressed in the cytoplasm by thioreductase-deficient (*trxB*) and glutathione reductase (*gor*) deficient strains (Terpe, 2006).

### Affinity tags and other fusion partners

Given the small size of bacteriocins, the incorporation of affinity tags, such as poly-His-tags, facilitates their detection and allows for one-step affinity purification. In addition, the use of fusion protein partners can increase the expression level, enhance protein solubility and assist in correct folding and disulfide bond formation (Ingham and Moore, 2007). Common fusion partners used for bacteriocin production include the cellulose binding domain (CBD_*cenA*_) (Klocke et al., 2005), maltose-binding protein (MBP) (Quadri et al., 1997; Kim et al., 2006), thiorredoxin (Trx) (Gibbs et al., 2004; Richard et al., 2004; Beaulieu et al., 2007; Yildirim et al., 2007; Jasniewski et al., 2008; Caetano et al., 2011a; Liu et al., 2011; Pal and Srivastava, 2014, 2015a,b; Jiang et al., 2016; Mustopa et al., 2016; Meng et al., 2017; Tang et al., 2018) and the small ubiquitin-related modifier SUMO (Wang et al., 2013).

Since secretion in the native hosts involves the cleavage of the signal sequence, such fusions often lack antimicrobial activity until chemical (e.g., cyanogen bromide cleaves proteins on the C-terminal side of methionine residues) or enymatic cleavage occurs. The cyanogen bromide (CNBr) chemical cleavage strategy has been used to release the mature PlnE and PlnF (Fimland et al., 2008) and PlnJ and PlnK (Rogne et al., 2009), as has also been described for production of carnobacteriocin B2 and BM1 (Jasniewski et al., 2008) and piscicolin 126 (Gibbs et al., 2004). However, the most common approach to release recombinant bacteriocins is to include a sequence, between the signal peptide and the bacteriocin, recognized by Factor Xa (Quadri et al., 1997; Kawai et al., 2003; Klocke et al., 2005; Moon et al., 2006; Ingham and Moore, 2007; Rogne et al., 2008; Shi et al., 2011), trypsin (Shi et al., 2012; Himes et al., 2016), thrombin (Klocke et al., 2005), enterokinases (Beaulieu et al., 2007; Jasniewski et al., 2008; Liu et al., 2011; Pal and Srivastava, 2014, 2015a,b; Jiang et al., 2016; Meng et al., 2016; Tang et al., 2018), and SUMO proteases (Wang et al., 2013). Alternatively, the use of intein fusions has been described for the cloning and expression of self-cleaving fusion forms of unmodified bacteriocins under appropriate buffer conditions (Ingham et al., 2005).

## *E. coli* strains for bacteriocin expression

Since bacteriocins can easily be degraded in the expression strain (Chen et al., 2012), *E. coli* BL21 (DE3) and its derivates are most frequently used for bacteriocin expression (Tables 1, 3) as they are deficient in the Lon protease and the outer membrane protease OmpT (Gottesman, 1996). Tuner™ (DE3) strains (Novagen) are *lacZY* deletion mutants of BL21. The lac permease mutation (*lacY*) allows uniform entry of IPTG into all cells in the population, which enables the regulation of the levels of protein expression by adjusting the concentration of the inductor IPTG. Other B related strains such as C41 (DE3) and C43 (DE3), described specially for the production of toxic proteins (Terpe, 2006), have been used successfully for the expression of the bacteriocin bovicin HJ50 while the use of *E. coli* BL21 (DE3) did not produce this peptide (Wang et al., 2016).

**Table 3:** Features of commercial *E. coli* strains commonly used for bacteriocin expression.

**Strain^a^**	**Strain background**	**lacI^q^**	***ompT*^−^**	***lon*^−^**	***trxB*^−^**	***gor*^−^**	***lacY^−^***	**Expression of toxic proteins**	**Rare codons tRNAs**	**pLysS**	**pLacI**	***dcm*^−^**	**Antibiotic resistance^b^**	**Supplier^c^**
AD494 (DE3)	K12	•			•								Kan	N
BL21	B		•	•								•		N
BL21 (DE3)	B		•	•								•		N
BL21 gold (DE3)	B		•	•								•	Tet	AT
BL21 (DE3) pLys	B		•	•						•		•	Cam	N
BL21 (DE3) RIL (DE3) pLysS	B		•	•					•	•		•	Cam	S
C41 (DE3) pLysS	B		•	•				•		•		•	Cam	L
C43 (DE3) pLysS	B		•	•				•		•		•	Cam	L
ER2566	K12		•	•								•		NEB
M15[pRep4]	K12	•											Kan	Q
Origami (DE3)	K12	•			•	•							Cam, Kan, Str, Tet	N
Origami (DE3) pLysS	K12	•			•	•				•			Cam, Kan, Tet	N
Rosetta (DE3) pLysS	B		•	•			•		•	•		•	Cam	N
Rosetta-gami 2 (DE3)	K12	•			•	•			•				Cam, Str, Tet	N
Tuner (DE3)	B		•	•			•					•		N
Tuner (DE3) pLacI	B		•	•			•				•	•	Cam	N

*lacI^q^ (constitutive expression of the lac repressor), ompT^−^ (mutation in outer-membrane protease), lon^−^ (Inactivation of Lon protease), trxB^−^ (mutation in thioredoxin reductase), gor^−^(mutation in gluthatione reductase), lacY^−^(lactose permease activity abolished), pLysS (encodes T7 lysozyme), pLacI (encodes lac repressor), dcm^−^(blocks cytosine methylation). Toxic proteins include many membrane proteins, some cytoplasmic proteins, and nucleases*.

a *Gold: provide increased transformation efficiency and produce high-quality miniprep DNA. RIL contain extra copies of the argU, ileY, and leuW TRNA genes*.

b *Antibiotic resistance: Cam, chloramphenicol; Kan, kanamycine; Tet, Tetracycline; Str, Streptomycin*.

c *Supplier: AT, Agilent Technologies; L, Lucigen; N, Novagen; NEB, New England Biolabs, Q, Quiagen, B, Stratagene*.

The designation DE3 indicates that the host is a lysogen of λDE3 which carries a chromosomal copy of the T7 RNA polymerase under the *lac*UV5 promoter required for the expression of genes under a T7 promoter (Rosano and Ceccarelli, 2014). To suppress the basal expression of T7 RNA polymerase prior to induction, especially important for toxic protein expression that affect cell growth and viability of the host, pLysS hosts carry a plasmid that encodes the T7 lysozyme, an inhibitor of T7 RNA polymerase. The pLacI designation is given to hosts bearing a plasmid that encodes the *lac* repressor.

K-12 derivatives have also been used for bacteriocin expression. *E. coli* JM109, which is *lon* protease deficient, has been used for gassericin A expression (Kawai et al., 2003). Divercin V41, enterocin CRL35, LSE_2163 and LSE_2386 and pediocin PA-1 that require disulphide bond formation for proper folding have been expressed in Origami™ (DE3) strains (Beaulieu et al., 2007; Yildirim et al., 2007; Kuo et al., 2013; Masias et al., 2014), which carry a double mutation in thioredoxin reductase (*trxB*) and glutathione reductase (*gor*) genes with an oxidative cytoplasmic environment that allows disulfide bond formation. The same *trxB/gor* mutations are carried by a derived Tuner™ strain, Origami B, combining the characteristics of BL21 and Origami hosts in one strain (Novagen). Other combinations, including Rosetta-gami™ (K-12 derivative) and Rosetta-gami™ B (BL21 derivative), facilitate the expression of eukaryotic proteins containing rare codons and cytoplasmic disulphide bond formation at the same time. Table 3 summarizes the features of commercial *E. coli* strains most used for bacteriocin production.

### Codon bias

Major differences between the codon usage of *E. coli* and the overexpressed protein may be an obstacle for bacteriocin production, especially in the case of rare codons. Rare codons are defined as codons used by *E. coli* at a frequency <1% (Kane, 1995), which means the tRNA for these codons are rare or lacking in the expression host. To overcome this problem, two strategies have been used: codon optimization of the mature sequences of bacteriocins (Richard et al., 2004; Ingham et al., 2005; Verdon et al., 2013) or increasing the availability of underrepresented tRNAs by supplying pRIL or pRARE plasmids. pRIL vector provides extra genes for the tRNAs for Arg (AGG/AGA), Ile (AUA), and Leu (CUA) while pRARE encodes tRNA genes for all the above-mentioned codons plus Gly (GGA) and Pro (CCC). pRIL or pRARE plasmids are supplied in the BL21 derivatives, BL21 (DE3) Codon Plus strain (Stratagene), and Rosetta (DE3) strains (Novagen), respectively. However, it is important to mention that although these strains can improve the level of production, they sometimes can cause a decrease in protein solubility (Rosano and Ceccarelli, 2014) or even decrease the growth rate of *E. coli* significantly (Masias et al., 2014).

### Selecting a suitable *E. coli* strain

**Figure 3:**
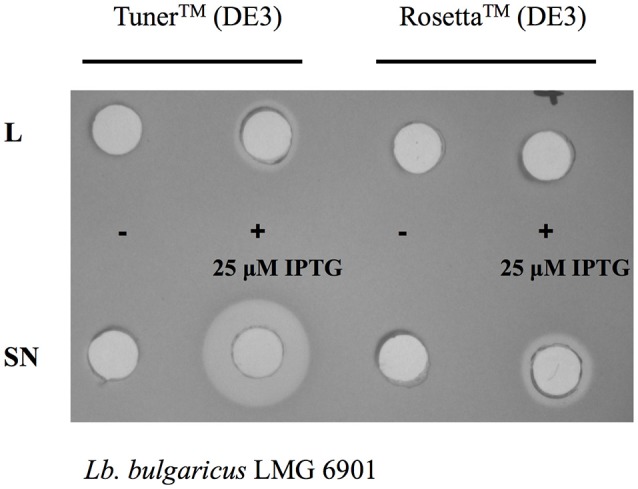
Differences in antimicrobial activity of cell-free supernatants (SN) and lysates (L) of *E. coli* Tuner™ (DE3) and Rosetta™ (DE3) cells carrying the plasmid encoding the whole operon of pediocin PA-1 without induction (−) and after 3 h of 25 μM IPTG induction (+).

Given that the expression level of bacteriocins can vary in different *E. coli* strains (Figure 3), it is recommended to test different strains, chosen to reflect the properties of the bacteriocin (e.g., disulfide bonds, rare codons, etc.) to select the best host for the heterologous expression. A clear example is described by Masias et al. (2014), who showed the expression of enterocin CRL35 in *E. coli* BL21, C41, C43, Origami, and Rosetta-gami 2. In this study, the expression of enterocin CRL35 was lower in *E. coli* C41 and C43 than in other *E. coli* strains. The best strains for enterocin CRL35 expression were *E. coli* Rosetta and *E. coli* Rosetta-gami 2 since they are able to synthesize proteins despite the presence of rare codons. In addition, this study showed that *E. coli* Rosetta produced an additional enterocin CRL35 variant. Therefore, the correct choice of the strain is critical for bacteriocin expression.

## Culture conditions for bacteriocin expression

Both culture media composition and culture conditions are important for optimizing the heterologous expression of bacteriocins and must be optimized for each bacteriocin in each expression system as reviewed in Table 1. Even two peptides of the same bacteriocin might require different conditions, for example the maximum soluble fraction of PLNC8α was observed under 0.5 mM IPTG induction for 16 h at 20°C, while for PLNC8β it was 0.2 mM IPTG for 20 h at 16°C (Jiang et al., 2016). Bacteriocin production can be also increased with different production strategies such as batch and fed-batch cultivation (Gibbs et al., 2004; Yildirim et al., 2007). Although it is hard to generalize, there are some observations that can be taken into account in terms of culture conditions to facilitate this trial-and-error process.

### Growth media

Luria Broth (LB) is the most commonly used medium for culturing *E. coli* for bacteriocin expression as it is easy to make and it is nutritionally rich. However, the cell density obtained with this medium is low, affecting bacteriocin yield. To overcome this problem, there are media superior to LB available for reaching higher cell densities such as 2 × yeast extract tryptone (YT), Terrific Broth (TB), and Super Broth (SB) (Rosano and Ceccarelli, 2014). 2 × YT medium has been used for expression of nukacin ISK-1 (Nagao et al., 2005) and epidermicin NI01 (Sandiford and Upton, 2012), while TB has been used for divercin V41 (Richard et al., 2004), carnobacteriocin Cbn1 and CbnB2 production (Jasniewski et al., 2008). Other bacteriocins have been produced using minimal medium (Rogne et al., 2008; Metelev et al., 2013). Therefore, there are no general rules. Pal and Srivastava (2015b) found higher Plantaricin E yield in LB than in TB, while Kuthning et al. (2015) found the best-producing conditions for Bliα and Bliβ lichenicidin peptides was medium M compared to 2 × YT, SB, TB, and LB-Kelly.

Alternatively, M9 medium supplementation with 10 mM of EDTA and 0.05% Tween 20 at the time of induction could also increase the final yield (Masias et al., 2014). In addition, the pH of the culturing media has also an impact on peptide yields either promoting bacteriocin expression or increasing peptide stability as described for lichenicidin expression which increased at pH 6.5 compared to pH 8 (Kuthning et al., 2015).

### Inducer agents

Bacteriocin expression levels can be tuned by varying the inducer concentration (Gutiérrez et al., 2005; Yildirim et al., 2007; Masias et al., 2014; Jiang et al., 2016), but use of a high concentration to fully induce the promoter does not necessarily lead to maximal expression due to the metabolic burden and toxicity of the inducer to the cells (Glick, 1995).

Although the IPTG inducible *lac* expression system of *E. coli* is the most used for bacteriocin expression, IPTG is expensive and toxic and therefore not suitable for large-scale production. This problem could be solved by replacing IPTG by lactose, which is not toxic, and has resulted in an increase in the production yield of some bacteriocins such as the carnobacteriocins Cbn BM1 and Cbn B2 (Jasniewski et al., 2008).

### Temperature

Temperature is one of the most important factors for the expression of functional proteins (Sambrook and Russell, 2001). Chen et al. (2012) showed that the expression level of sakacin P was higher when *E. coli* BL21 (DE3) carrying pET28a-sakP was induced at 20°C than at 37°C. Similar results were observed when the expression of plantaricin E was induced at 25°C rather than at 37°C (Pal and Srivastava, 2015b). Although the optimum growth temperature for *E. coli* is around 37°C, the cell growth and the protein synthesis are slowed down at lower temperatures, which provides the peptides the time and optimal environment to fold into their native conformation (Sambrook and Russell, 2001; Peng et al., 2004), decreasing the aggregation and increasing the expression of soluble protein. In addition, lowering the temperature, in combination with the time after induction, might shift the codon usage bias in *E. coli* sufficiently to solve some codon-usage based expression problems (Terpe, 2006). Therefore, it is essential to determine the optimal induction temperature in each case to improve the amount of the soluble fraction. When inclusion bodies formation is a problem, it is recommended to express the protein in the range of 15–25°C (Rosano and Ceccarelli, 2014).

## Conclusion

The development of heterologous expression systems to improve bacteriocin yield may facilitate their characterization and broaden their applications in food and pharmaceutical industries. Currently *E. coli* is the most popular recombinant protein expression platform. However, choosing the perfect combination of expression vector and strain for bacteriocin production in *E. coli* is not possible *a priori* due to the many variables that can affect bacteriocin production. This review covers different strategies used for the bacteriocin expression in *E. coli* to help the process of choosing the best expression system and the conditions for any particular bacteriocin with a view to producing bacteriocins economically for both food and pharmaceutical applications.

## Author contributions

BM-P, MR, PC, CH, and RR wrote the manuscript and approved its final version.

### Conflict of interest statement

The authors declare that the research was conducted in the absence of any commercial or financial relationships that could be construed as a potential conflict of interest.
